# Prevalence of Klinefelter syndrome among infertile men with spermogram abnormalities: a cross-sectional study at a referral laboratory in Rabat, Morocco

**DOI:** 10.11604/pamj.2026.54.36.50134

**Published:** 2026-06-08

**Authors:** Zahra Takki Chebihi, Elbekkay Chadli, Fatine Hadrya, Bouchra Belkadi, Aziza Belkhayat

**Affiliations:** 1 Laboratory of Microbiology and Molecular Biology, Faculty of Sciences, Mohammed V University, Avenue Ibn Battouta, BP 1014, Rabat 10000, Morocco,; 2 Biolab Laboratory, Rabat, Morocco,; 3 Department of Cytogenetics, Institut Pasteur, Casablanca, Morocco,; 4 High Institute of Health Sciences, University Hassan First, Settat, Morocco,; 5 Faculty of Sciences, University Ibn Tofail, Kenitra, Morocco

**Keywords:** Klinefelter syndrome, male infertility, azoospermia, oligospermia, chromosome aberrations, Morocco

## Abstract

**Introduction:**

Klinefelter syndrome (KS), the most common genetic cause of male infertility, remains vastly underdiagnosed in Morocco and across Africa due to limited cytogenetic access.

**Methods:**

we conducted a 5-year retrospective cross-sectional study (2020-2024) at BIOLAB Laboratory, Rabat, analyzing 415 infertile Moroccan men (18-58 years) with spermogram abnormalities (azoospermia or oligospermia) who were referred for karyotyping. Standard R-banding cytogenetics (≥15 metaphases; ISCN 2020) was performed on peripheral blood lymphocytes.

**Results:**

among 415 infertile men included in the study, Klinefelter syndrome was identified in 45 cases (10.8%). The prevalence of KS was higher among azoospermic men (12.6%) compared with oligospermic men (3.1%). Most cases were non-mosaic 47, XXY (91.1%), while mosaic forms accounted for 8.9% of cases.

**Conclusion:**

Klinefelter syndrome was frequently observed among infertile men with severe spermogram abnormalities in this referral-based population, particularly among azoospermic patients. These findings highlight the importance of cytogenetic evaluation in infertile men presenting with azoospermia or severe oligospermia.

## Introduction

Infertility affects approximately 10-15% of couples globally, with male factors contributing to nearly half of cases [1,2]. While multifactorial in origin-encompassing hormonal, environmental, and lifestyle, influences genetic causes account for ~15% of male infertility overall and up to 30% in men with azoospermia [3,4]. Among genetic etiologies, Klinefelter syndrome (KS) stands out as the most common sex chromosome aneuploidy in men (1 in 600) and a leading genetic cause of severe male infertility [5,6].

Klinefelter syndrome results from supernumerary X chromosome material, most commonly 47, XXY-arising from meiotic nondisjunction in parental gametogenesis [7,8]. Despite X-chromosome inactivation, ~15% of X-linked genes escape silencing, leading to dysregulation of key spermatogenic pathways, germ cell depletion, and testicular degeneration [9,10]. This manifests clinically as hypergonadotropic hypogonadism, azoospermia (in ~90% of non-mosaic cases), reduced muscle mass, tall stature, and, in some, neurocognitive challenges [11-15]. Klinefelter syndrome is found in 8-12% of azoospermic men, ~6% of oligospermic men, and 3-4% of all infertile men globally [16,17].

Despite its high prevalence, KS remains profoundly underdiagnosed: estimates suggest up to 65% of affected individuals are never identified, largely due to subtle or variable phenotypes, low clinical suspicion, and especially in resource-limited regions-limited access to cytogenetic testing [18,19]. Karyotype analysis remains the gold standard for diagnosis [20,21], and early detection enables timely interventions – such as testosterone replacement, fertility preservation (e.g., sperm cryopreservation), and genetic counseling – that significantly improve metabolic, psychological, and reproductive outcomes [22-24].

In Morocco and across North Africa, no published epidemiological data on KS exist, and national infertility guidelines rarely mandate routine karyotyping. This gap impedes early diagnosis, delays care, and obscures the true burden of KS in a region where genetic services are often centralized and inaccessible to rural populations [25-28]. This study aimed to estimate the prevalence and describe the cytogenetic characteristics of Klinefelter syndrome among infertile men with spermogram abnormalities referred for karyotyping at BIOLAB Laboratory, Rabat, Morocco.

## Methods

**Study design and setting:** we conducted a retrospective cross-sectional study at Biolab Laboratory, a private medical genetics facility in Rabat, Morocco. The study was carried out over five years, from January 2020 to December 2024, and involved the analysis of cytogenetic data from infertile men referred for karyotype evaluation.

**Study population:** the study population comprised infertile men aged ≥18 years referred for cytogenetic evaluation because of abnormal spermogram results or clinical suspicion of Klinefelter syndrome. We included all consecutive patients presenting with azoospermia, oligospermia, or clinical features suggestive of Klinefelter syndrome who underwent karyotype analysis during the study period. Patients without spermogram abnormalities or with incomplete cytogenetic data were excluded. As this was a referral-based descriptive study, no formal sample size calculation was performed.

**Data collection:** data were retrospectively extracted from laboratory records and clinical files. Peripheral blood samples were collected for conventional cytogenetic analysis using standard lymphocyte culture techniques. R-banded karyotyping was performed, with analysis of at least 15 metaphases per patient, and karyotypes were interpreted according to the International System for Human Cytogenomic Nomenclature (ISCN 2020).

**Definitions:** azoospermia was defined as the complete absence of spermatozoa in semen samples, and oligospermia as a sperm concentration below 15 million/mL, per World Health Organization 2021 criteria. Klinefelter syndrome was defined by the presence of an additional X chromosome in males, including non-mosaic (47, XXY) and mosaic forms.

**Statistical analysis:** data were analyzed using Microsoft Excel and IBM SPSS Statistics. Continuous variables are presented as medians and interquartile ranges, and categorical variables as frequencies and percentages. Analyses were descriptive, focusing on prevalence estimation.

**Ethical considerations:** the study was approved by the Ethics Committee for Biomedical Research of the Faculty of Medicine and Pharmacy of Rabat (Reference No. 10/25, dated 26 February 2025). Written informed consent was obtained from all participants prior to cytogenetic testing, and data were anonymized to ensure confidentiality.

## Results

**Characteristics of the study population:** a total of 415 infertile men referred for constitutional karyotyping at BIOLAB Laboratory (Rabat, Morocco) between 2020 and 2024 met the inclusion criteria and were included in the analysis. The median age was 35 years (interquartile range: 31-40; range: 18-58). The largest age group was 31-40 years, representing 242 patients (58.3%) (Figure 1). Based on referral indications, 270 patients (65.1%) presented with azoospermia, 95 (22.9%) with oligospermia, and 50 (12.0%) were referred because of clinical suspicion of Klinefelter syndrome (KS) without documented severe sperm abnormalities (Figure 2). The prevalence of KS was 12.6% among azoospermic men, 3.2% among oligospermic men, and 16.0% among patients referred because of clinical suspicion of KS. The frequency of spermogram abnormalities and the prevalence of KS according to referral category are summarized in Table 1. Cytogenetic analysis identified Klinefelter syndrome as the most frequent chromosomal abnormality in the study population. A representative R-banded karyotype showing a non-mosaic 47, XXY constitution in an azoospermic patient is presented in Figure 3. Chromosome analysis and nomenclature were performed according to the International System for Human Cytogenomic Nomenclature (ISCN 2020).

**Figure 1:**
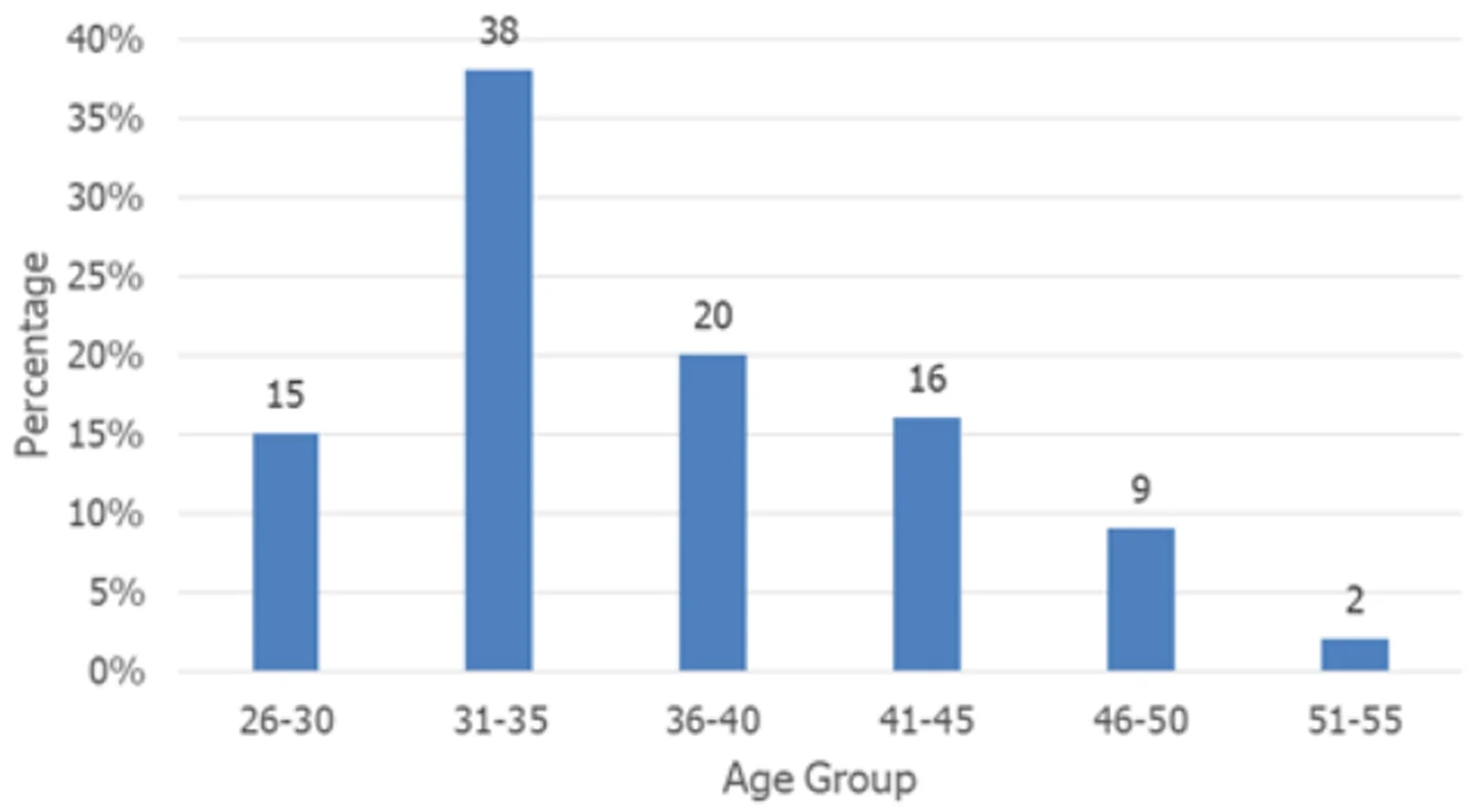
age distribution of the study population

**Figure 2:**
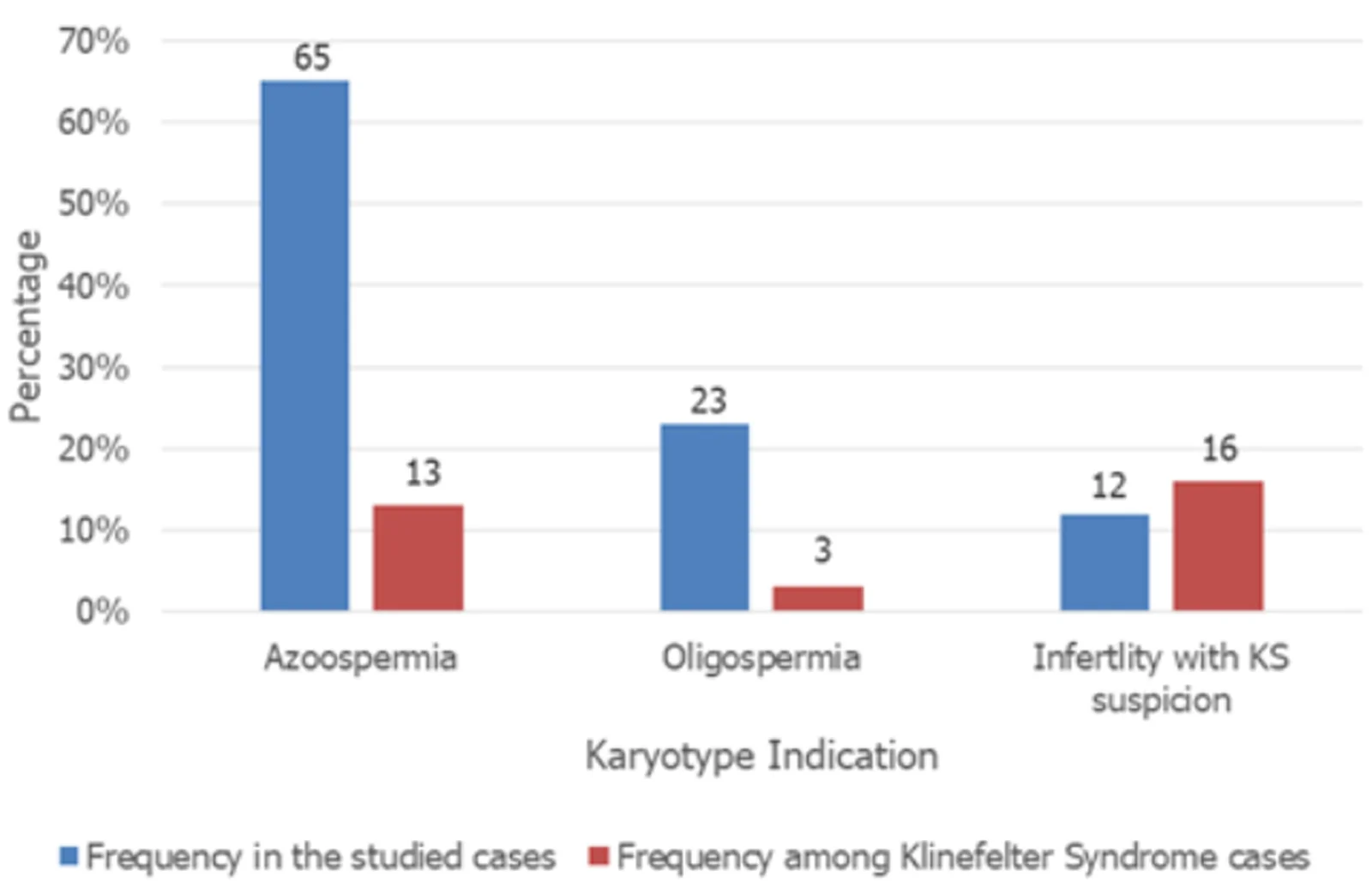
distribution of spermogram abnormality categories in the study population

**Table 1:** frequency of spermogram abnormalities and prevalence of Klinefelter syndrome among 415 infertile Moroccan men referred for constitutional karyotyping, BIOLAB Laboratory, Rabat, Morocco, 2020-2024

Indication/spermogram abnormality	Number of cases	Percentage of the total (n=415)	Number among KS cases	Percentage of KS cases (n=45)	Prevalence of KS reported in the literature for each group
Azoospermia	270	65.0%	34	12.6%	8-12%
Oligospermia	95	22.9%	3	3.2%	6%
Infertility with KS suspicion	50	12.0%	8	16.0%	-

KS: Klinefelter syndrome

**Figure 3:**
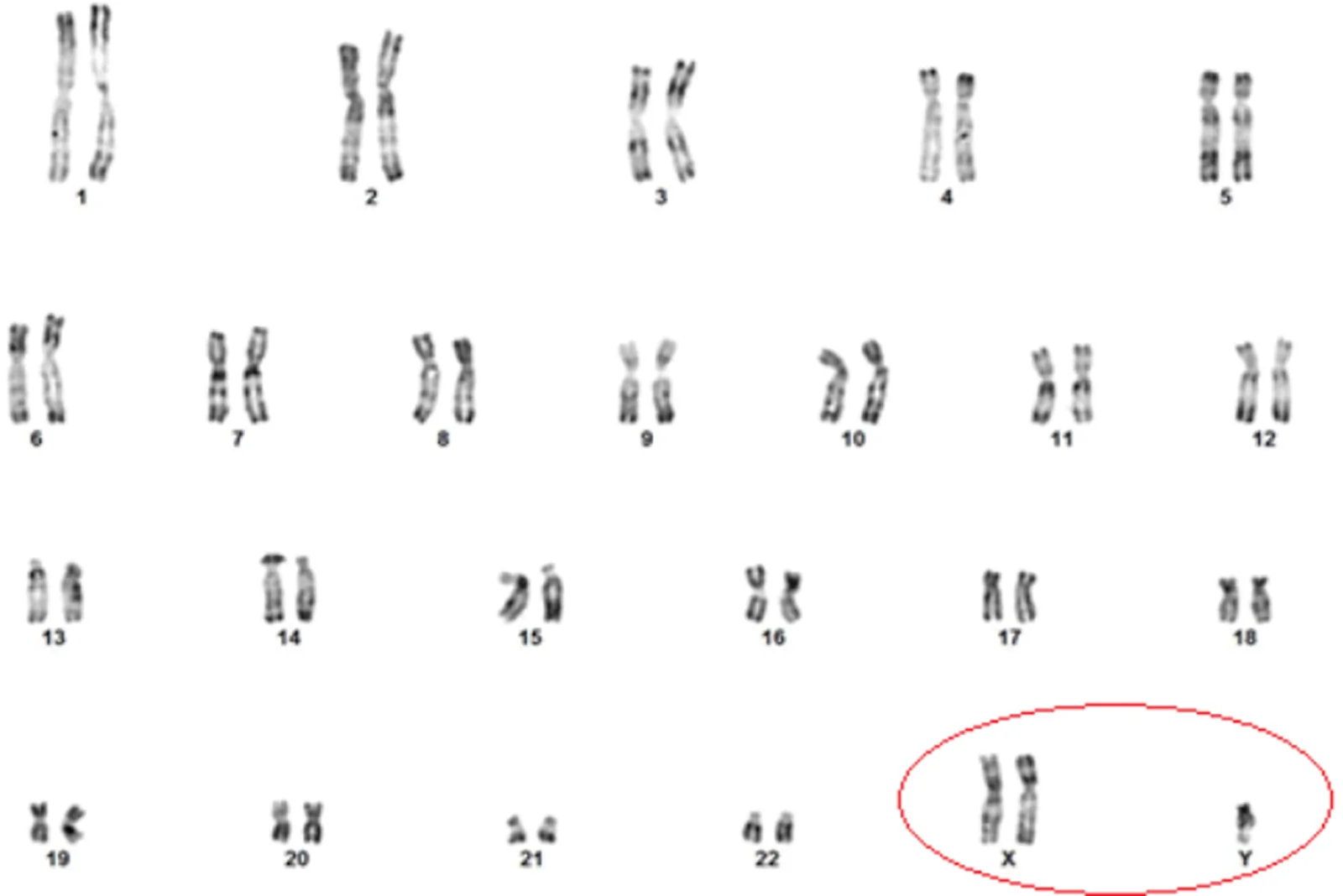
representative r-banded karyotype of a non-mosaic 47, XXY Klinefelter syndrome case

**Prevalence of Klinefelter syndrome:** Klinefelter syndrome was identified in 45 of the 415 patients, corresponding to an overall prevalence of 10.8%. Among azoospermic men, KS was diagnosed in 34 of 270 cases (12.6%). Among oligospermic men, 3 of 95 patients were diagnosed with KS (3.2%). Among the 50 men referred due to clinical suspicion of KS, cytogenetic confirmation was obtained in 8 cases (16.0%). The prevalence of KS according to spermogram category is presented in Table 1.

**Cytogenetic patterns of Klinefelter syndrome:** among the 45 patients diagnosed with Klinefelter syndrome (KS), the non-mosaic 47, XXY karyotype was the predominant cytogenetic pattern, observed in 41 cases (91.1%), whereas mosaic forms (46, XY/47, XXY) were identified in 4 cases (8.9%). These proportions are consistent with published data, which indicate that approximately 85-90% of KS cases are non-mosaic and 10-15% are mosaic. Azoospermia was the most common sperm abnormality among KS patients, affecting 34 of 45 cases (75.6%), followed by oligospermia in 3 cases (6.7%), while 8 patients (17.8%) were referred based on clinical suspicion of KS. Notably, all mosaic KS cases were observed in azoospermic patients. The distribution of cytogenetic patterns is presented in Table 2.

**Table 2:** distribution of mosaic and non-mosaic Klinefelter syndrome among 45 diagnosed cases in 415 infertile Moroccan men, BIOLAB Laboratory, Rabat, Morocco, 2020-2024

Forms of KS	Number of cases	Percentage of KS cases (n=45)	Prevalence of KS forms reported in the literature
Non-mosaic	41	91.1%	85-90%
Mosaic	4	8.9%	10-15%

KS: Klinefelter syndrome

## Discussion

This study aimed to estimate the prevalence of Klinefelter syndrome (KS) among infertile Moroccan men with spermogram abnormalities referred for karyotyping at BIOLAB Laboratory, Rabat. We found that KS was diagnosed in 45 of 415 men (10.8%), with a notably high prevalence of 12.6% among azoospermic men and confirmed non-mosaic 47, XXY as the dominant form (91.1%) [26]. All four mosaic KS cases presented with azoospermia, reinforcing its role as a critical clinical indicator for cytogenetic evaluation [27].

Our finding of 12.6% KS prevalence among azoospermic men is slightly higher than the widely cited global range of 8-12% [5], and exceeds recent reports from India (9.2%) [16]. It aligns, however, with upper-end European series (up to 14%) [14], suggesting potential regional variation in KS burden or referral patterns. While no prior epidemiological data exist for Morocco, a Tunisian cytogenetic study reported sex chromosome anomalies – predominantly 47, XXY – in azoospermic men, indicating a likely similar pattern across North Africa [28]. The elevated prevalence in our sample may reflect increased genetic susceptibility, environmental influences, or heightened referral bias toward severe cases in a specialized genetics center [20].

Notably, all mosaic KS cases (46, XY/47, XXY) in our study were azoospermic, despite literature suggesting milder phenotypes and occasional fertility in mosaic individuals [23]. This discrepancy may stem from the well-documented limitation that blood-derived karyotypes do not always reflect testicular mosaicism [29]. It is possible that the post-zygotic error occurred early enough to affect the germ cell lineage disproportionately, leading to complete spermatogenic failure even in the presence of a euploid somatic cell line [26]. Regardless of mechanism, our data confirm that azoospermia remains a highly sensitive marker for KS – even in mosaic forms – and should trigger routine karyotyping in clinical practice [25].

Klinefelter syndrome arises from an extra X chromosome, most commonly 47, XXY, due to meiotic nondisjunction [16,26]. Although X-chromosome inactivation silences most genes, approximately 15% escape inactivation, leading to dysregulation of spermatogenic pathways [30-32]. Key escapee genes – including RBMY, DAZ, SHOX, ZFX, and TSPYL2 [33-38] – alongside androgen receptor dysfunction, disrupt germ cell development and Sertoli – Leydig cell function, ultimately causing testicular degeneration and azoospermia [35,14]. These molecular mechanisms explain the near-universal association between KS and severe sperm abnormalities observed in our cohort [26].

These findings carry significant clinical implications for Morocco and similar settings. Early diagnosis enables timely testosterone replacement [39], psychological support [5], metabolic monitoring [20], and fertility preservation through sperm cryopreservation or MicroTESE-ICSI [40,41]. Given the interchromosomal effect and potential transmission risks, preimplantation genetic testing and genetic counseling are essential components of reproductive management [42,43]. Integrating karyotyping into national infertility guidelines for all azoospermic men would address the current diagnostic delay - evidenced by a median diagnosis age of 35 years - and reduce the lifelong burden of undiagnosed KS [23,44].

This single-center study has important strengths, including standardized cytogenetic protocols (≥15 metaphases, ISCN 2020 compliance, dual verification), complete data capture over five years, and the first structured KS prevalence estimate from Morocco. However, limitations include potential selection bias, as participants were clinically referred rather than population-based, which may overrepresent severe cases. Hormonal profiles and physical measurements (e.g., testicular volume) were inconsistently available, and limited access to genetic services in rural areas may have led to underrepresentation of certain patient groups. Despite these constraints, our findings provide a crucial foundation for future multi-center studies and policy development across the Maghreb.

## Conclusion

This study demonstrates that Klinefelter syndrome is a frequent chromosomal abnormality among infertile men referred for cytogenetic evaluation in Morocco, with an overall prevalence of 10.8% and a higher prevalence among azoospermic patients (12.6%). The non-mosaic 47, XXY karyotype was the predominant cytogenetic pattern, and azoospermia was the most common sperm abnormality observed among affected individuals. All four mosaic cases also presented with azoospermia. The median age at diagnosis was 35 years, reflecting delayed identification. These findings confirm KS as a significant contributor to severe male infertility in this clinical setting and highlight azoospermia as a key indicator warranting cytogenetic evaluation. The high prevalence supports the need for increased clinical suspicion and access to karyotyping in similar referral centers across North Africa.

### What is known about this topic

- Klinefelter syndrome is a common chromosomal cause of male infertility, particularly associated with azoospermia and severe oligospermia;
- Most men with Klinefelter syndrome remain undiagnosed due to variable clinical presentation and limited access to cytogenetic testing;
- The non-mosaic 47, XXY karyotype accounts for the majority of Klinefelter syndrome cases worldwide.

### What this study adds

- Provides the first structured epidemiological data on Klinefelter syndrome among infertile men referred for cytogenetic evaluation in Morocco;
- Shows a high prevalence of Klinefelter syndrome among azoospermic men (12.6%) in a referral-based Moroccan clinical setting;
- Confirms the predominance of the non-mosaic 47, XXY karyotype and its strong association with severe sperm abnormalities.
